# A cellular genetics approach identifies gene-drug interactions and pinpoints drug toxicity pathway nodes

**DOI:** 10.3389/fgene.2014.00272

**Published:** 2014-08-29

**Authors:** Oscar T. Suzuki, Amber Frick, Bethany B. Parks, O. Joseph Trask, Natasha Butz, Brian Steffy, Emmanuel Chan, David K. Scoville, Eric Healy, Cristina Benton, Patricia E. McQuaid, Russell S. Thomas, Tim Wiltshire

**Affiliations:** ^1^Division of Pharmacotherapy and Experimental Therapeutics, UNC Eshelman School of Pharmacy at the University of North Carolina at Chapel HillChapel Hill, NC, USA; ^2^The Hamner Institutes for Health Sciences, Research Triangle ParkNC, USA; ^3^Meredith CollegeRaleigh, NC, USA

**Keywords:** high throughput screening, mouse model, *in vitro* screening, high-content imaging, gene-drug interaction, genome-wide association

## Abstract

New approaches to toxicity testing have incorporated high-throughput screening across a broad-range of *in vitro* assays to identify potential key events in response to chemical or drug treatment. To date, these approaches have primarily utilized repurposed drug discovery assays. In this study, we describe an approach that combines *in vitro* screening with genetic approaches for the experimental identification of genes and pathways involved in chemical or drug toxicity. Primary embryonic fibroblasts isolated from 32 genetically-characterized inbred mouse strains were treated in concentration-response format with 65 compounds, including pharmaceutical drugs, environmental chemicals, and compounds with known modes-of-action. Integrated cellular responses were measured at 24 and 72 h using high-content imaging and included cell loss, membrane permeability, mitochondrial function, and apoptosis. Genetic association analysis of cross-strain differences in the cellular responses resulted in a collection of candidate loci potentially underlying the variable strain response to each chemical. As a demonstration of the approach, one candidate gene involved in rotenone sensitivity, *Cybb*, was experimentally validated *in vitro* and *in vivo*. Pathway analysis on the combined list of candidate loci across all chemicals identified a number of over-connected nodes that may serve as core regulatory points in toxicity pathways.

## Introduction

Toxicology and toxicity testing are in the midst of a transformation. A series of expert panels, workshops, and strategic reviews have proposed a transition from an apical endpoint-based evaluation of chemical and drug safety to a focus on identifying key molecular initiating events and pathway perturbations leading to adverse effects (Woodruff et al., 2008; Firestone et al., 2010; Berg et al., 2011; Silbergeld et al., 2011; Keller et al., 2012). The proposed transition is being driven by the need to reduce the cost and time associated with evaluating the safety of drugs and chemicals, to allow broader coverage of compounds, mixtures, endpoints and life-stages in the evaluation, and to provide a more robust basis for risk assessment through the identification and application of mechanistic data (National Research Council. Committee on Toxicity Testing and Assessment of Environmental Agents, 2007). Within the National Research Council (NRC) report, the initiating event and subsequent pathway perturbation were collapsed into the term, toxicity pathway, and this term was explicitly defined as “cellular response pathways that, when sufficiently perturbed, are expected to result in adverse health effects” (National Research Council. Committee on Toxicity Testing and Assessment of Environmental Agents, 2007).

The sequencing of multiple mammalian genomes, including both mouse (Waterston et al., 2002) and human (Venter et al., 2001), has led to a series of new approaches using genetics to identify the mode-of-action leading to adverse effects of certain drug or chemical exposures. In particular, inbred mouse strains provide a unique resource for toxicogenetic studies. They have extensive, publicly-available genotype data, enabling genome-wide association analysis on phenotypic differences identified following drug or chemical treatment. In an example of the application of inbred mouse strains to evaluate mode-of-action, Harrill and colleagues demonstrated an association between a variant of the orthologous human gene, *CD44*, and acetaminophen hepatotoxicity (Harrill et al., 2009). Genetic approaches to chemical and drug toxicity have also been applied *in vitro* using lymphoblastoid cell lines from the Centre d'Etude du Polymorphisme Humain (CEPH) panel (Peters et al., 2009; O'Shea et al., 2011; Watson et al., 2011a,b; Lock et al., 2012).

In the present study, we described the development and application of a complementary high-throughput cellular genetics platform to experimentally identify genetic determinants of chemical toxicity and define potential core nodes in toxicity pathways. Primary mouse embryonic fibroblasts (MEFs) from 32 inbred mouse strains were treated in concentration-response format with 65 diverse environmental and pharmaceutical compounds. Phenotypic parameters of cell health status were measured using high-content imaging and genetic association analysis of cross-strain differences in the cellular responses was used to identify candidate genes underlying such differences. The results of the study suggest that the screening approach can identify genes involved in the cytotoxic response to each chemical and that pathway analysis on the combined list of candidate genes across all chemicals may identify common toxicological response regulatory nodes.

## Materials and methods

### Compounds

A total of 65 compounds were evaluated—26 were ToxCast Phase I compounds, 27 were pharmaceutical compounds, and 12 were mechanistic compounds with known modes-of-action (Data Sheet 1). The ToxCast compounds were chosen based on early cytotoxicity screening results from the ToxCast program as well as a diversity in *in vivo* adverse responses for comparative data analyses. The pharmaceutical compounds were chosen to include drugs known to be involved in a range of adverse reactions (e.g., drug-induced liver injury) and those generally considered safe (e.g., aspirin and ibuprofen), for a comprehensive assessment of cellular toxicity profiles. The mechanistic compounds were chosen to provide a core set of putative cellular toxicity pathways. Each compound was screened at nine concentrations that ranged from 15 to 100 uM, facilitating concentration-response analyses. All compound stock solutions and subsequent dilutions were prepared in DMSO, with the exception of ibuprofen, which was diluted in water. Master 384-well microplates were prepared for each compound using the layout provided in Presentation 1. Three-fold compound serial dilutions were prepared using a Biomek 2000 (Beckman Coulter, Brea, CA), and the final concentration ranges are listed in Data Sheet 1. A separate microplate was prepared with 8.33 mM of valinomycin solution for positive control wells; final concentration was 33 μM.

### Cell culture

Primary MEF cells from 32 inbred mouse strains were purchased as custom isolations (P0 cultures combined from multiple 12.5 day-old embryos) from The Jackson Laboratory (Bar Harbor, ME). A minimum of 6 embryos were used for each strain. The selected inbred mouse strains were a subset of strains from the Mouse Phenome Panel (Bogue and Grubb, 2004) and included the 129S1/SvImJ, A/J, AKR/J, BALB/cByJ, BTBR *T*^+^ *tf*/J (currently named BTBR *T*^+^ *Itpr3^tf^*/J), BUB/BnJ, C3H/HeJ, C57BL/6J, C57BR/cdJ, C57L/J, CBA/J, CE/J, CZECHII/EiJ, DBA/2J, FVB/NJ, I/LnJ, KK/HlJ, LG/J, LP/J, MRL/MpJ, NOD/LtJ, NON/LtJ, NOR/LtJ, NZO/HlLtJ, NZW/LacJ, PL/J, RIIIS/J, SEA/GnJ, SJL/J, SM/J, SWR/J, and WSB/EiJ strains of mice. Prior to use in the study, MEFs from each strain were expanded from P0 to P3, frozen in cryotubes containing 10% DMSO (Sigma-Aldrich, Milwaukee, WI), and stored in liquid nitrogen.

To prepare for screening, the MEFs from each strain were thawed and cultured in tissue-culture-treated, filter cap CELLSTAR flasks (Greiner Bio-One, Monroe, NC) using modified DMEM growth media, (Cellgro, Manassas, VA) containing 10% v/v fetal bovine serum (Cellgro, Manassas, VA), 1% v/v non-essential amino acid solution, (Sigma-Aldrich, Milwaukee, WI), and 1% v/v penicillin/streptomycin solution (Sigma-Aldrich, Milwaukee, WI). The cells were maintained in a humidified environment at 37°C and 5% CO_2_. Due to some strain variation in growth rates, the thawing and plating of the MEF cells was staggered to achieve ~90% density at the time of screening.

### High content screening

On the day of plating, MEF cells were washed with phosphate-buffered saline (PBS, Gibco-Life Technologies, Grand Island, NY), trypsinized (1X Trypsin-EDTA solution, Sigma-Aldrich, Milwaukee, WI), harvested, and the concentration of the cell suspension was determined using an Invitrogen Countess automated cell counter (Invitrogen, Carlsbad, CA). The cells were diluted in complete media to a concentration of 5 × 10^4^ cells/ml and then seeded into 384-well, PDL-coated microplates using Multidrop 384 dispensers (Thermo Scientific, Waltham, MA). Every microplate contained 12 wells for each of the 32 strains (Presentation 1). Two different microplates were used in the screening protocol (Perkin Elmer View plates, PDL-coated, Cat #. 6007718; Aurora 200 μM film bottom with COP polymer; Cat #. 32411). Cells were plated at two different densities: 1500 cells/well for plates assigned to the 24 h time point and 1000 cells/well for the 72 h time point. For both time points the final volume of cell culture media per well was 50 μl. The plates were incubated overnight at 37°C and 5% CO_2_ to allow for cell attachment.

**Figure 1:**
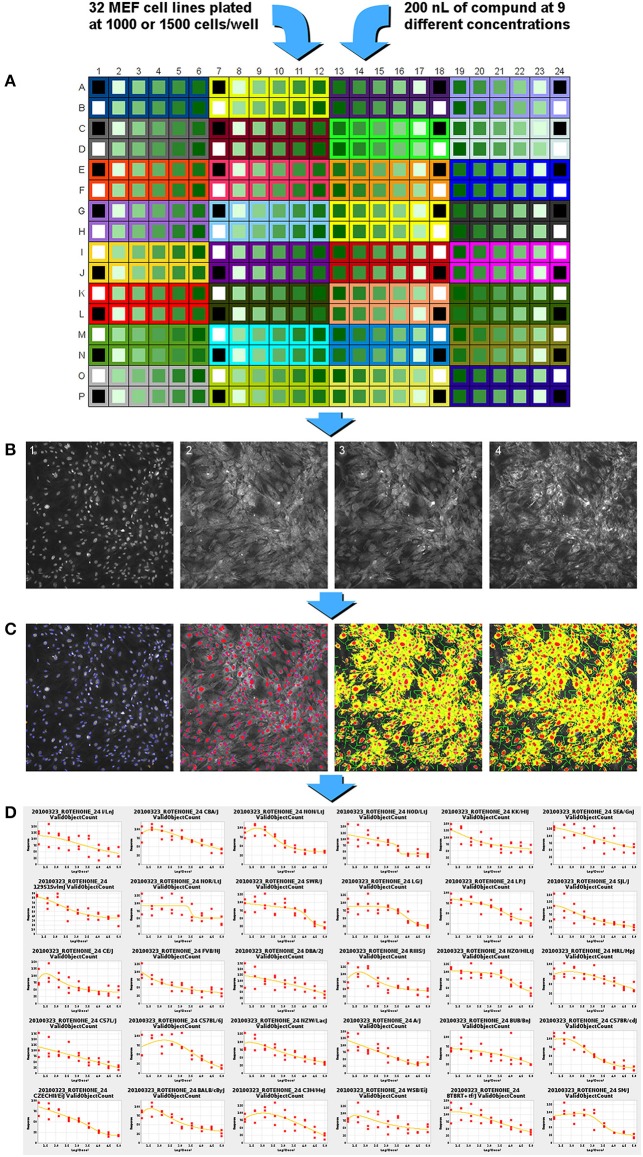
**Experimental flow chart for high-content screening of the MEF lines in concentration-response format across 65 diverse compounds. (A)** MEF cells from 32 inbred strains are plated into a single 384 well plate. Compound is added from a master plate using a 200 nl pin-tool resulting in 9 concentrations of compound (15 nM to 100 μM for most chemicals, Data Sheet 1 contains the different ranges used), one positive control compound and two wells of negative control, DMSO control, and no treatment control. **(B)** Cell staining reagents are added, incubated, and then imaged with a high-content imaging system. Image 1: nuclei staining with Hoechst 33342; image 2: membrane permeability dye channel; image 3: mitochondrial membrane potential dye; image 4: cytochrome C antibody staining. **(C)** Images are analyzed and segmented using the Cellomics vHCS Toolbox Compartmental Analysis bioapplication. **(D)** Dose-response curves generated for each assay endpoint. EC_50_ to EC_80_ or EC_120_ to EC_150_ values are generated in 5-point intervals for each assay per strain.

The cells were treated with compound following the incubation period by transfer of 200 nl of compound stock solution from the master 384-well microplates using a pin tool (VP 384FP1CB Pin tool with FP3NS200H pins, V&P Scientific, San Diego, CA) and a Biomek FX system (Beckman Coulter, Brea, CA). Triplicate plates were treated for each compound and time point. Among the 12 wells for each MEF cell line on each plate, 9 wells were used for the concentration response of the compound of interest (15–100 uM); one well was treated with an equivalent volume of vehicle (DMSO or water); one well was treated with a positive reference compound (33 μM); and one well did not receive any treatment (Figure 1A). For the compounds where DMSO was used as vehicle, the final concentration of DMSO was 0.4%.

After 24 or 72 h of treatment, cells were labeled and processed using a modified Cellomics Multiparameter Cytotoxicity 3 Kit (Thermo Scientific, Rockford, IL). The protocol was adapted from a 96-well plate assay to a 384-well plate assay. The kit contained Hoechst 33342 nucleic acid dye, a cell and nuclei permeability dye, a mitochondrial membrane potential dye, a primary monoclonal cytochrome c antibody matched with secondary DyLight-649 conjugated goat anti-mouse IgG antibody, wash buffers, permeabilization buffer, and blocking buffer. Briefly, 13 μl/well of Live Cell Staining Solution (containing mitochondrial membrane potential dye and cell permeability dye) was added to each well and the cells were incubated at 37°C for 30 min. The cells were then fixed with 21 μl/well of 16% paraformaldehyde for 20 min at room temperature. Following fixation, the solution was removed and the cells were washed with 50 μl/well wash buffer. The cells were then permeabilized with 25 μl/well permeabilization buffer for 10 min at room temperature. The cells were washed twice with 50 μl/well wash buffer. The cells were blocked with 25 μl/well blocking buffer for 15 min at room temperature. The blocking buffer was removed and 13 μl/well of the primary antibody solution was added. The cells were incubated for 60 min at room temperature. The cells were washed three times with 50 μl/well wash buffer and then incubated for 60 min with 13 μl/well of the secondary antibody solution. The cells were washed again three times with 50 μl/well wash buffer. An additional 50 μl/well of wash buffer was added to the cells and sealed for scanning. All washing steps were performed using a BioTek ELx405 Select Microplate Washer (BioTek, Winooski, VT).

Plates were delivered to a BD Pathway 435 high-content imaging instrument (BD Biosciences, Franklin Lakes, NJ) equipped with 200 W metal halide lamp (89 North, Burlington, VT), using a Twister II plate handling robot (Perkin Elmer, Hopkinton, MA). Accurate focusing and acquisition of cell images was accomplished using laser-based auto-focusing. Excitation (ex), dichroic mirror (dm), and emission (em) wavelengths are expressed in nanometers units, and approximate exposure value (ev) times to achieve at least 25% signal saturation are expressed as seconds for the following probes: Hoechst 33342 (ex 377/dm 409/em 435/ev 0.014); cell permeability dye (ex 482/dm 506/em 536/ev 0.008); mitochondria potential probe (ex 543/dm 562/em 593/ev 0.012); and cytochrome c—DL649 (ex 628/dm 660/em 692/ev 0.25). A pixel array size of 2784 × 1536 from a 4 × 3 tile montage of acquired images were captured using 2× CCD camera binning through an Olympus 20× / 0.75 NA objective lens (Figure 1B). All images were saved in BD Attovision software in native file format (TIF). The images were manually reviewed for quality to identify: (1) fluorescent artifacts; (2) images that failed to focus; (3) absence of cell objects in image; and (4) wells with poor algorithmic fit of image. Images showing any of these qualities were removed from the analysis.

The image files passing the quality check were renamed using automated Perl script commands to satisfy nomenclature for using the Thermo Scientific Cellomics vHCS Toolbox Compartmental Analysis bioapplication algorithm. Camera input dimensions equal to 2784 × 1536 were programmed into the vHCS Toolbox for interpretation of all plate well images. The bioapplication algorithm measurement of pixel size, shape, and intensity of cell objects from each fluorescent probe in the wells were calculated based on segmentation and thresholding of individual objects. Each cell nuclei of Hoechst 33342 label intensity was used to identify and collectively enumerate cell objects per image in the scannable area in the well; doublets and small objects outside of threshold values were rejected. The primary well level summary values extracted from the images were the number of nuclei (cell loss); mean average fluorescence intensity of the mitochondrial membrane potential dye in the cytoplasm area (mitochondrial function); mean average fluorescent intensity of cytochrome c in the cytoplasm (apoptosis); and the mean average fluorescence intensity of the permeability dye within the nuclear area (membrane permeability) (Figure 1C).

### Concentration-response analysis and quantitative trait loci (QTL) mapping

**Figure 2:**
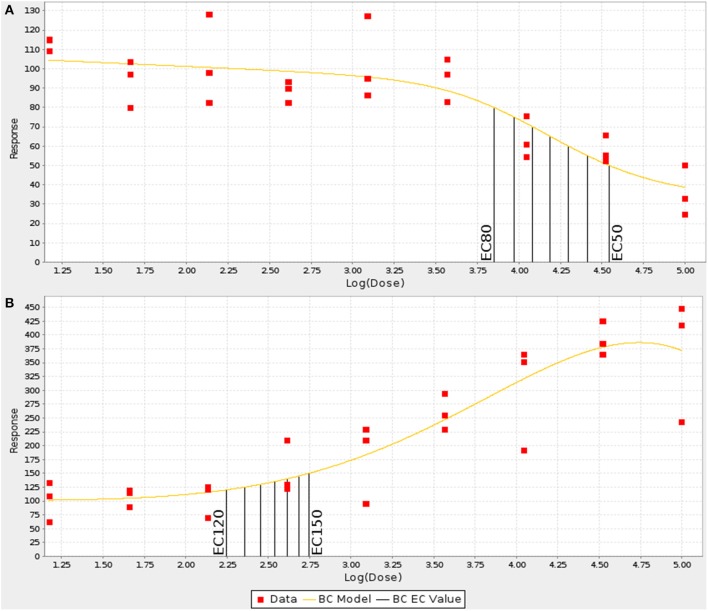
**Examples of increasing and decreasing concentration response curves**. The concentration response curves for each endpoint were fit using a Brain-Cousens model and EC_*n*_ values were interpolated at defined intervals. **(A)** For decreasing responses, EC_50_–EC_80_ were calculated in 5% steps. **(B)** For increasing values, EC_120_–EC_150_ were calculated in 5% steps.

For each strain, the numerical values generated by the image analysis bioapplication algorithm were normalized using the value obtained for the vehicle only well of the respective plate. Concentration-response curves were generated for each MEF line by fitting the normalized data in triplicate to the Brain-Cousens model (Brain and Cousens, 1989). The reason the Brain-Cousens model was used as opposed to more traditional models (e.g., Hill model) was its ability to accommodate both monotonic and non-monotonic concentration response curves since a significant number of treatments and MEF lines showed non-monotonic concentration-response behavior. The fitting was performed within a custom Java application using the drc package (version 2.0-1; http://www.bioassay.dk) for R (version 2.10.1; http://www.r-project.org) (Figure 1D). In cases where the curve fitting failed due to data points that were clearly outliers, they were manually removed. A maximum of four points were removed from a single curve (from 27 total points in a curve: nine doses in triplicates); the total number of data points removed among the 32 strains for each drug-endpoint pair is presented as Data Sheet 2. For endpoints showing decreasing response values with increasing concentrations (cell loss and mitochondrial function), the curves were then used to calculate individual EC_50_ up through EC_80_ values in 6 stepwise increments for each of the strains (i.e., EC_50_,EC_55_,EC_60_,EC_65_,etc.) (Figure 2A). For endpoints showing increasing response values with increasing concentrations (membrane permeability and apoptosis), EC_120_ up through EC_150_ were calculated in 6 stepwise increments for each strain (Figure 2B).

Genome-wide association (GWA) mapping was performed for each of the EC_*n*_ values from each assay endpoint and time point using the SNPster algorithm (McClurg et al., 2007). This approach defines genomic loci that associate with the phenotype pattern and are termed quantitative trait loci. Values used in the association analysis can be found in Data Sheet 3. Compounds were used for genetic analysis only if a minimum of 25 strains had at least three interpolated EC_*n*_ values, thus excluding the compounds with no or minimal observed effect in the concentration-response curves. The selected GWA approach uses the haplotype structure inferred from overlapping 3-SNP windows. An F-statistic is calculated from the phenotype values grouped according to the different haplotypes by a One-Way ANOVA test; *p*-values are estimated by bootstrapping the phenotypic values, 1 × 10^6^ bootstraps were used, giving SNPster a maximum -log(*p*-value) score of 6.0 (McClurg et al., 2007). The SNP genotypes used for SNPster were obtained from the Mouse Diversity Array set (Yang et al., 2009), which are available from the CGDSNPdb website (http://cgd.jax.org/cgdsnpdb/). The identification of QTLs for each endpoint was done by selecting the top 2% SNPs by -log(*p*-value) of each EC_*n*_ and then averaging the -log(*p*-value) of all EC_*n*_ at each SNP position. Genomic regions with mean -log(*p*-value) greater than or equal to 3.5, including a window of 100 kb on each side, were selected for further analysis. The region between two SNPs with a mean -log(*p*-value) above or equal to a threshold of 3.5 was included if the SNPs were within less than 1 Mb of each other. Candidate quantitative trait genes (QTG) that completely or partially overlap with the regions associated with drug response were selected for validation or network analysis. To restrict the downstream analyses to genes more likely to be relevant to the cellular responses in MEFs, only genes with known expression in MEFs were used. Expression levels were measured in MEFs from six inbred strains of mice (A/J, AKR/J, C3H/HeJ, C57BL/6J, CBA/J, DBA/2J) using the Affymetrix Mouse Genome 430 2.0 Array (Affymetrix, Santa Clara, CA). Genes were considered expressed if the expression level, after data processing with the gcRMA algorithm, was greater than a value of 50 for at least one of the strains.

### Network analysis

Due to the relatively small number of candidate genes identified for each endpoint, the candidate genes were combined for network analysis. A full list of candidate genes and their associated endpoints are listed in Data Sheet 4. The identification of over-connected network nodes was conducted using the MetaCore database (Version 6.12 build 42289, GeneGo, St. Joseph, MI). The *p*-values associated with the overconnection analysis were calculated based on a hypergeometric distribution. The *p*-value is the probability of randomly obtaining the observed size of intersection between the candidate gene list and the network or pathway from the MetaCore database. The *p*-values were corrected for multiple comparisons using false discovery rate (FDR; Benjamini and Hochberg, 1995).

### Candidate gene *in vitro* validation

The gene chosen for experimental validation was selected based on literature evidence of biological relevance, expression level in MEFs, and correlation between EC values and expression levels in other tissues (spleen, liver, and adipose tissues). A gene would only be considered for validation if is expressed in MEFs. In this manuscript, we present the validation of *Cybb* as a candidate gene for variable sensitivity in cell loss following rotenone treatment using *in vitro* knockdown and overexpression approaches as an example to show the utility of the toxicogenetic screen for gene identification.

C57BL/6J MEFs were electroporated using the Amaxa system (MEF1 Nucleofector Kit with the T-20 program; Lonza, Basel, Switzerland). A total of 2 × 10^6^ cells were used per electroporation. Overexpression was achieved using pCMV-SPORT6 vectors containing the *Cybb* cDNA (MMM1013-9498866; Open Biosystems, Lafayette, CO). Cells were co-transfected with 3 μg of *Cybb* pCMV-SPORT6 and 2 μg of pmaxGFP plasmid (Lonza, Basel, Switzerland). Electroporation efficiency was determined by flow cytometry to be 40%. Knockdown was performed using siRNA oligonucleotides (sc-5827; Santa Cruz Biotechnology, Santa Cruz, CA). An siRNA oligonucleotide that does not specifically target any known cellular mRNA was used as control (sc-45924; Santa Cruz Biotechnology, Santa Cruz, CA). Transfected cells were plated in 96-well plates. Eight replicate wells were used for each rotenone concentration in the overexpression experiment, and three replicates were used in the knockdown experiment. Cells electroporated with the overexpression vector were incubated for 24 h prior to treatment, and cells transfected with siRNA oligonucleotides were incubated for 48 h before treatment to allow for a reduction in the amount of the targeted proteins in the cells. Cells were treated with rotenone in concentration-response format from 0.13 to 100 μM. Treated cells were, after 24 and 72 h, fixed with 4% paraformaldehyde in PBS and stained with Hoechst 33342. Fixed cells were imaged with a Cellomics ArrayScan VTI HCS Reader (Thermo Scientific, Rockford, IL). A total of 10 fields were captured in each well using a 10X objective. Images were analyzed using the Cellomics Compartmental Analysis HCS BioApplication (Thermo Scientific) to determine the cell number at each concentration.

Transfected cells were also used for mRNA isolation to confirm the overexpression and knockdown of the genes at the same time points. Total cell RNA was isolated using the RNeasy Mini Kit (Qiagen, Hilden, Germany) followed by a reverse transcription reaction using Superscript II Reverse Transcriptase (Invitrogen, Carlsbad, CA) and random hexamers. Quantitative PCR (qPCR) was performed in duplicate using SYBR Green (Thermo Scientific, Rockford, IL; forward primer: ACACTGACCTCTGCTCCTGAG, reverse primer: TCTTCACTGGCTGTACCAAAG) on a CFX96 real-time PCR system (Bio-Rad, Hercules, CA). Fold-change in gene expression was determined using the ΔΔ*Ct* method with the *B2m* gene as an endogenous control (forward primer: TTCTGGTGCTTGTCTCACTGA, reverse primer: CAGTATGTTCGGCTTCCCATTC).

### Candidate gene *in vivo* validation

**Figure 3:**
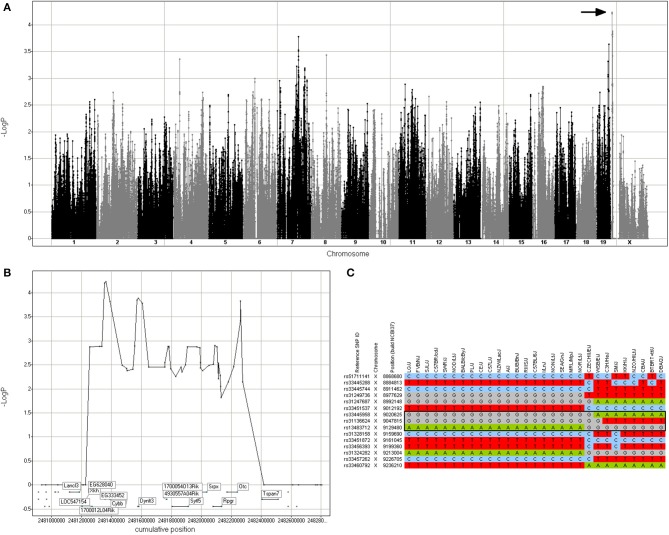
**Genomic region associated with rotenone cytotoxicity. (A)** GWA Manhattan plot for cell loss after 72 h of rotenone treatment. The region with the highest -log *p*-value that was selected for further analysis is indicated by the arrow. **(B)** Detail of the region in chromosome X annotated with the candidate genes. **(C)** Haplotype structure of the inbred mouse strains in the region. The black box indicates the 3-SNP haplotype with the best SNPster association score, which includes the *Cybb* gene (chromosome X 9,012,380–9,046,450).

Based on the haplotypes present in the chromosome X region associated with rotenone cellular response, two mouse strains were selected for *in vivo* validation. FVB/NJ and BTBR *T*^+^ *tf*/J (SNPster GGA and ATG haplotypes, respectively; Figure 3). All animal experiments were conducted on approved protocols under the direction of the UNC Institutional Animal Care and Use Committee (IACUC). All *in vivo* experiments were performed using male mice, 9–10 weeks of age. Animals were subjected to a treadmill endurance test after repeated exposure to rotenone. Twelve mice from each strain were obtained from The Jackson Laboratory (Bar Harbor, ME). Mice were maintained on a 12:12 h light/dark cycle and given water and food *ad lib*. Mice received daily IP injections of vehicle (*n* = 6) or 0.05 mg/kg of rotenone (Sigma-Aldrich) diluted in olive oil (*n* = 6) from 0800 to 1200 for 14 days. Body weight was measured and recorded daily from 0800 to 1200 for each mouse. After study treatment, mice were trained on an Exer-3/6 Open Treadmill apparatus (Columbus Instruments, Columbus, Ohio) for three consecutive days for 17, 18.5, and 21.5 min, respectively. Following training, mice were tested on Day 4 for endurance at the same time as the training days (1400–1800). Mice were placed individually on an Exer-3/6 treadmill lane and forced to exercise via an electrical stimulus grid attached to the treadmill. The final test was conducted on a 5° inclination with gradual acceleration from 0 to 8 m/min for the first 120 s. Then, speed was slowly increased at 2 m/min intervals within 30 s after mice maintained their speed to 12 m/min and then to 14 m/min for 900 s each. Mice were forced to run on a final speed of 16 m/min. The test was terminated individually, according to the mouse's endurance. A criterion of 10 shocks was set as a termination marker for testing.

## Results

### Phenotype measurements

A high-content imaging assay was used to evaluate the cellular health status of the individual MEF lines after treatment with 65 compounds. The design of the study allowed MEFs from all 32 inbred mouse strains to be accommodated on a single 384-well plate and treated with a single compound in 9-point concentration-response curves with positive and negative controls for each strain (Figure 1). The simultaneous culture, treatment, and immunofluorescent staining of the 32 cell lines on the same plate minimizes batch effects across strains that could potentially confound the genetic analysis of the phenotypes. Since a wide range of compounds with unknown toxic effects on MEFs was tested, two incubation times were used; cells were treated for 24 and 72 h and three replicate plates were used for each time point. Four endpoints were measured at both tested time points: cell loss, mitochondrial function, apoptosis, and membrane permeability. For each strain, a comparison between vehicle only and no vehicle wells did not show a significant difference in phenotype values, suggesting that a 0.4% DMSO concentration did not cause cellular toxicity. Based on the cell loss endpoint, 35 of the 65 compounds tested showed some cytotoxicity in the concentration range tested, reaching the EC_80_ threshold. Across all endpoints, 38 compounds had a detectable cellular response, reaching either the EC_80_ or EC_120_ thresholds (Data Sheet 5).

### Genetic association analysis

The experimental design used in the study allowed each individual plate and associated replicates to be treated as a single unit for the genetic analysis on each of the four endpoints and the two time points. Although 38 compounds exhibited variable cellular phenotype responses, not all of these responses were appropriate for genetic analysis. To obtain a robust GWA, at least 25 or more mouse strains were required to have at least three EC_*n*_ values. This ensures that the concentration range used was appropriate and high enough to induce a cytotoxic response. The use of a combined -log(*p*-value) from different EC_*n*_ minimizes the effect of curve slope variations in the final loci selection. Using this criteria, a total of 28 compounds had genomic regions associated with a phenotypic response as defined by having a combined mean -log(*p*-value) of ≥3.5 for at least one endpoint-time point pair. For these compounds, a total of 196 genomic regions were identified, with 538 unique RefSeq genes (547 total genes) mapped to 114 of the regions. A list of the compound-time point pairs linked with a putative QTL (-log *p*-value ≥3.5) as well as the QTGs for the different endpoints is provided as supplementary material (Data Sheet 4).

### Candidate gene validation

To demonstrate that the approach can identify valid genetic modifiers, a candidate gene was selected for functional validation. The cytotoxic response to rotenone exhibited robust phenotypic changes that were variable across the different mouse strains. A 1.1 Mb locus on Chromosome X (8.91–10.02 Mb) was identified from genetic analysis, and this locus contained 10 candidate genes. Among the 10 candidate genes, *Cybb* (aka *Nox2*) had a strain distribution pattern that mirrored the haplotype structure at the locus peak (Figure 3). *Cybb* has been traditionally thought of as a component of the microbicidal oxidase system of phagocytes, but it is also expressed in other cell types such as neurons, cardiomyocytes, skeletal muscle myocytes, hepatocytes, endothelial cells, and hematopoietic stem cells (Bedard and Krause, 2007; Anilkumar et al., 2008). *Cybb* is also expressed in MEFs (data not shown).

**Figure 4:**
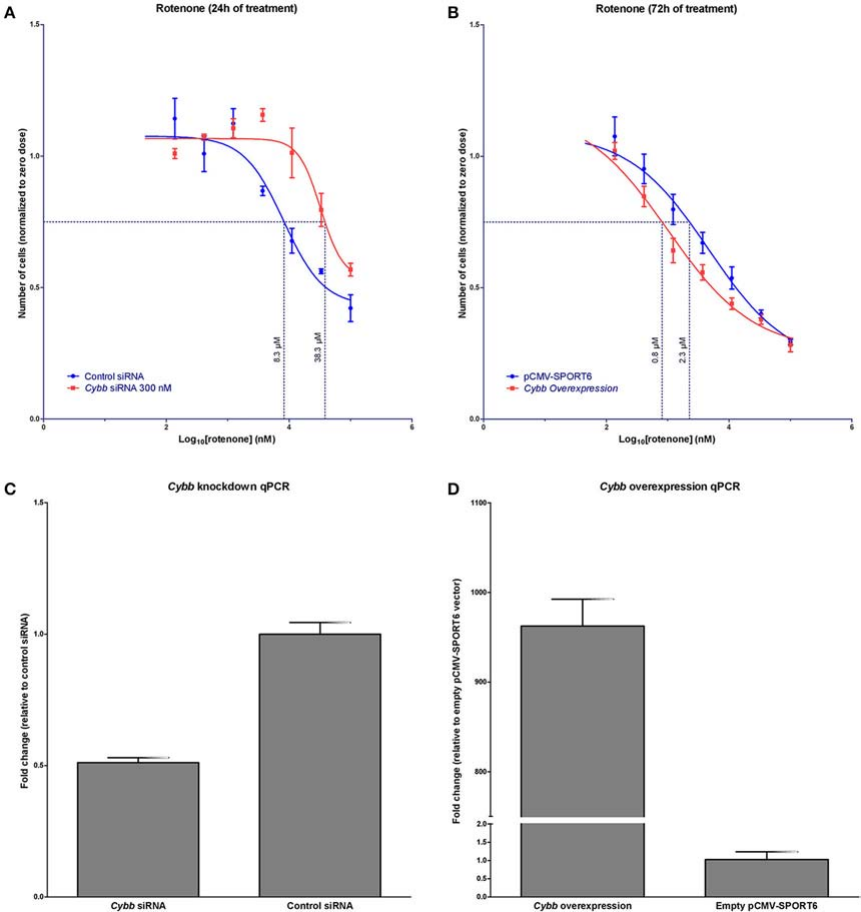
***In vitro* validation of *Cybb* in rotenone cytotoxicity. (A)** Concentration response of rotenone cytotoxicity following siRNA knockdown of *Cybb* (*n* = 3 replicates at each concentration). Knockdown of the *Cybb* gene shifts the concentration-response curve to the right. Dotted line indicates the EC_75_. **(B)** Concentration response of rotenone cytotoxicity following *Cybb* over-expression (*n* = 8 replicates at each concentration). Over-expression of *Cybb* shifts the concentration-response curve to the left. The dotted line shows the EC_75_. **(C)** Observed expression change in *Cybb* with siRNA knockdown (*n* = 4 replicates), and **(D)** over-expression (*n* = 2 replicates). Data are expressed as means ± *SE*.

It was hypothesized that changes in *Cybb* expression would affect rotenone *in vitro* toxicity. Indeed, the knockdown of *Cybb* in C57BL6/J MEFs using siRNA resulted in a shift of the rotenone concentration-response curve to the right, EC_75_ shifted from 8.3 μM (95% CI [4.9, 14.6]) to 38.3 μM (95% CI [26.8, 62.2]). *Cybb* over-expression in this same cell line, using a cDNA plasmid, shifted the concentration response curve to the left, changing the EC_75_ from 2.3 μM (95% CI [1.4, 3.4]) to 0.8 μM (95% CI [0.6, 1.1]) (Figures 4A–D).

**Figure 5:**
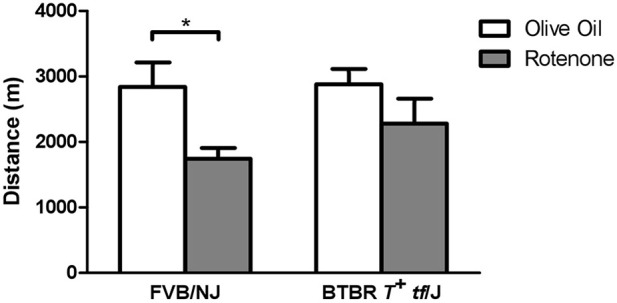
***In vivo* validation of *Cybb* in rotenone toxicity using the treadmill exhaustion test**. Two inbred mouse strains were tested for distance traveled during a 3.5 h time span in the stress treadmill test. The mice treated with vehicle (*n* = 6 per strain) showed similar distances for the two strains; however, FVB/NJ mice treated with rotenone (*n* = 6) had a larger performance decrease than BTBR *T*^+^ *tf*/J mice (*n* = 6) ^*^p < 0.05. Data are expressed as means ± *SE*.

To evaluate the role of different *Cybb* haplotype groups in rotenone toxicity *in vivo*, two mouse strains with different haplotypes were tested for maintenance of aerobic capacity in the treadmill endurance test following repeated treatment with rotenone. FVB/NJ mice (GGA haplotype group) showed a larger performance deficit in the treadmill endurance test after rotenone treatment compared to the BTBR *T*^+^ *tf*/J mice (ATG haplotype group) (Figure 5). Only the FVB/NJ strain showed a significant difference between rotenone- and control-treated mice (*p* = 0.02). On average, FVB/NJ vehicle-treated mice ran 1096 m more than rotenone-treated FVB/NJ mice.

### Pathway enrichment and network analysis

Due to the relatively low number of QTGs associated with each endpoint, the candidate genes for all chemicals were combined across all four endpoints prior to pathway enrichment analysis. The enriched pathways consisted primarily of those relating to G-protein signaling, cytoskeletal remodeling, and oxidative stress (Table 1). Under the assumption that while the candidate genes involved in the phenotypic differences may be distributed over a wide range of pathways, there may exist an underlying set of nodes that are connected to multiple candidate genes and represent the core of a network driving the adverse cellular responses. A network analysis was performed and six over-connected nodes were identified (Table 2).

**Table 1:** **Pathway enrichment analysis for combined set of candidate genes**.

**Pathway**	**Candidate genes in pathway/Total genes**	**FDR**
G-protein signaling_Rap1A regulation pathway	4/40	4.3E-02
G-protein signaling_Cross-talk between Ras-family GTPases	3/23	6.8E-02
Cytoskeleton remodeling_RalA regulation pathway	3/30	7.3E-02
G-protein signaling_G-Protein alpha-q signaling cascades	3/34	7.3E-02
Oxidative stress_Role of ASK1 under oxidative stress	3/34	7.3E-02
G-protein signaling_RhoA regulation pathway	3/34	7.3E-02

**Table 2:** **Over-connected nodes in network analysis of combined set of candidate genes**.

**Node**	**Protein function**	**Number of interactions**	***p*-value^a^**
*Myc*	Transcription factor	59	4.5E-06
*Tnfrsf1a*	Receptor	9	3.5E-04
*Cdk1*	Kinase	18	4.0E-04
*Prkacb*	Kinase	16	6.4E-04
*Ppp2ca*	Phosphatase	9	2.0E-03
*Inppl1*	Phosphatase	4	5.1E-03

a *All over-connected nodes significant at FDR < 0.1*.

## Discussion

Reports such as the NRC Toxicity Testing in the twenty-first Century (National Research Council. Committee on Toxicity Testing and Assessment of Environmental Agents, 2007) have suggested performing toxicity testing of chemicals and drugs using *in vitro* assays based on toxicity pathways. Identifying and characterizing these pathways was listed in the NRC report as one of the critical areas of knowledge development. In this study, we describe an approach that combines *in vitro* screening with genetic approaches for the experimental identification of genetic loci modifying cellular responses to individual chemicals as well as potential core nodes in putative toxicity pathways when applied across large numbers of chemicals. The key development in this screening method is the use of a well-characterized genetic model that allows variable phenotype responses to be measured and GWA analysis to identify potential QTG. The genetic intervals defined using this approach are small enough to be able to identify and test candidate genes for validation of association.

Recent advances in high-throughput screening technologies have allowed researchers to test thousands of perturbations in parallel in a cost-effective process. Specific application of high-content imaging to *in vitro* toxicity testing maintains many advantages as it permits multiplexed measurements of multiple endpoints and the evaluation of subtle cytological changes in response to chemical or drug treatment. High-content imaging has been used to predict drug-induced liver injury using cultured human hepatocellular carcinoma cells (HepG2), where the observed cytotoxicity endpoints were highly concordant with human hepatotoxicity (O'Brien et al., 2006).

*In vitro* toxicity studies for the purpose of genetic analysis have relied mostly on the use of immortalized human cell lines. Several groups have employed CEPH lymphoblastoid cell lines to identify genomic regions and genes that are associated with both pharmaco- and toxicogenetic responses (Huang et al., 2008; Bleibel et al., 2009; Peters et al., 2009; Watson et al., 2011a,b; Lock et al., 2012). The use of immortalized cell lines in these studies provide considerable experimental convenience compared to primary cells, but significant factors cannot easily be controlled in immortalized cell lines. These factors include a dysfunctional apoptotic and cell cycle control mechanism, undefined or abnormal genetic backgrounds acquired through mutations accumulated with long-term tissue culture, and a lack of control of the immortalization process (Marshak and Greenwalt, 2007; Welsh et al., 2009). The use of human induced pluripotent stem cells have been proposed as an alternative to toxicity testing, as these cells can be expanded in culture and differentiated into the cell types of interest; however, additional technical developments are needed to make this possible (Scott et al., 2013).

The use of mouse cells in *in vitro* assays can overcome some of the shortcomings associated with human cell-based systems. For example, mouse models allow for easier access to primary cells and experiments are more easily reproduced due to the genetic stability of inbred strains. The existence of genetic information for a large number of inbred mouse strains and the availability of high-quality primary cells enable GWA studies based on a cellular genetics approach. Inbred mouse strains also make possible to perform QTG mapping; the strains are genetically and phenotypically diverse and have a high number of recombinations, which improves mapping resolution (McClurg et al., 2006). Mice have been used successfully in toxicogenetic studies with results that were translatable to humans (Guo et al., 2006; Harrill et al., 2009; Zhang et al., 2011) and are routinely used in safety evaluation of drugs and other chemicals. Although toxicity in rodents and humans is discordant in some cases (Shanks et al., 2009), a relatively small number of inbred mouse strains can be used to model the wide genetic and phenotypic variability found in human populations (Paigen and Eppig, 2000), enabling high-throughput screens that capture a broad range of response variance. Additionally, the capacity to identify genes that modify the response to chemicals at a cellular level can still provide insights into human toxicity mechanisms. Cytotoxicity studies using cells derived from inbred mouse strains have been performed (Watters et al., 2003), but not in a high-throughput manner.

In our experimental set-up we proposed to broadly measure aspects of cell health as we had selected a wide range of chemicals to test in this generalized assay. We intended to show that even with a general approach you could screen multiple compounds and identify specific gene-drug associations, and importantly we hypothesized that there would be some “common” toxicity pathways that are highlighted even when multiple compounds with very different toxicity profiles were screened. We demonstrated that it is possible to map QTL using high-content imaging data from MEFs collected from an inbred mouse diversity panel and treated with multiple chemicals and pharmaceutical compounds. We identify candidate genes of interaction for multiple drugs, and we selected one of these for more extensive validation. For one of the treatments, a candidate gene, *Cybb*, was associated with rotenone cytotoxicity. *Cybb* encodes for gp91^phox^, a subunit of NADPH oxidase. Previous studies have demonstrated that rotenone binds to gp91^phox^ and activates NADPH oxidase to induce superoxide production (Gao et al., 2003; Zhou et al., 2012; Pal et al., 2014). The siRNA knockdown of *Cybb* was consistent with an expected reduction in superoxide production by rotenone exposure, leading to increased cell survival and a right shift of the concentration-response curve. Conversely, the over-expression of *Cybb* was consistent with increased superoxide production, reduced cell survival, and a shift the concentration-response curve to the left. These results are in concordance with the neurotoxic effects of rotenone observed in gp91^phox^ knockout mice compared to wild-type animals (Gao et al., 2003). From a broader perspective, the validation results demonstrated that a cellular genetics approach can be used to identify genes involved in the *in vitro* toxicological effects of specific chemicals.

Translating the results from *in vitro* studies in embryo fibroblasts to *in vivo* responses may not always be straightforward. In our particular case, increasingly high concentrations of rotenone led to cell death *in vitro*. If given at equivalent *in vivo* doses, a large amount of cell death may result in organ failure or lethality, but the relevance of these high concentration effects would be questionable. In order to examine potentially more relevant *in vivo* effects of rotenone treatment, we hypothesized that repeated sub-lethal doses of rotenone would result in reduced aerobic capacity and lead to decreased performance in the treadmill endurance test based on the role of gp91^phox^ in cellular respiration. When low dose of rotenone was administered daily over the course of 14 days, the FVB/NJ mouse strain showed a greater performance deficit after rotenone treatment compared to the BTBR *T*^+^ *tf*/J mouse strain, as we predicted from cell-based studies. Although there are no *Cybb* non-synonymous SNPs among the selected set of strains, multiple SNPs in the coding and 3′-UTR regions follow the same haplotype distribution pattern as the one that was associated with rotenone response, which suggests that these SNPs could potentially have an effect on the amount of protein. Based on the *Cybb* expression profile from the inbred strains in several different tissues (for example, the strain expression profile of *Cybb* in adipose tissue and the EC_50_ response of MEFs to rotenone is highly correlated with a *p*-value < 8.95 × 10^−5^), we hypothesize that the differential expression of *Cybb* between strains is the likely cause of the differential response to rotenone. These studies demonstrate that a cell-based QTL mapping result could lead to identification of genetic variants that are relevant to whole-organism toxicological responses.

To evaluate the candidate genes across multiple chemicals, pathway enrichment analysis was performed on the candidate genes combined across endpoints and treatments. The majority of candidate genes were distributed over a range of pathways resulting in a limited number that were statistically enriched. The wide distribution may be due to a number of factors including the false positive genes present in the list, the diversity of pathways involved in the integrated cellular responses, or the diversity in toxicological modes-of-action across the chemicals evaluated. In each case, spreading the candidate genes across a range of pathways would diminish the statistical enrichment of any one putative toxicity pathway. Those pathways that were enriched could be considered toxicity pathways and consisted primarily of those relating to G-protein signaling, cytoskeletal remodeling, and oxidative stress. Of those enriched, a previous study has linked inhibition of RhoA signaling to adriamycin-induced cardiomyopathy (Wang et al., 2011). Other studies have linked ASK1 signaling to acetaminophen-induced apoptosis in the liver (Niso-Santano et al., 2010), angiotensin II-induced cardiac injury (Nako et al., 2012), 1-methyl-4-phenyl-1,2,3,6-tetrahydropyridine (MPTP) toxicity (Lee et al., 2012), troglitizone-induced hepatocellular injury (Lim et al., 2008), and other adverse responses.

Due to the low number of significantly enriched pathways, network analysis was performed to identify over-connected nodes among the candidate genes. The rationale for performing this type of analysis was that even though the candidate genes may be spread across a range of canonical signaling pathways, there may exist a set of core nodes connecting the genes that represent critical junctions in toxicity networks. Among the nodes identified, Myc was highly over-connected among the candidate genes. Myc is a well-known transcription factor with a global influence in the cell transcriptome, and it plays key roles in cell growth and apoptosis (Dang et al., 2006). Likewise, Tnfrsf1a is a cellular receptor that plays a role in cell growth, death, and stress response (Chen and Goeddel, 2002). Cdk1 is a catalytic subunit of the M-phase promoting factor (MPF) and is essential for G1/S and G2/M phase transitions of the cell cycle (Rhind and Russell, 2012). Prkacb is a cAMP-dependent protein kinase that has diverse effects on cellular function (Daniel et al., 1998). Some of these functions include regulation of proto-oncogenes (Wu et al., 2002), regulating cellular localization of signaling proteins and receptors (Higuchi et al., 2003), and altering stability of nuclear receptors (Yum et al., 2009). Ppp2ca is one of the four major Ser/Thr phosphatases and it plays a key role in many critical cellular processes through the dephosphorylation of signaling molecules such as Akt, p53, Myc, and β-catenin (Seshacharyulu et al., 2013). Finally, Inppl1 (aka Ship2*)* is involved in the regulation of insulin function (Vinciguerra and Foti, 2006) and also plays a role in the regulation of epidermal growth factor receptor turnover (Zwaenepoel et al., 2010), and actin remodeling (Venkatareddy et al., 2011). In each case, the over-connected genes are associated with critical cellular functions that when excessively perturbed will likely lead to adverse effects.

In summary, we demonstrated that genetically characterized primary cell lines from multiple inbred mouse strains can be utilized within *in vitro* toxicology assays. Inbred mouse strain panels are widely used to detect the genetic contributions to complex diseases and other phenotypes; here, we show the use of this valuable model population in a flexible and scalable *in vitro* toxicogenomic pipeline. The variable cellular responses among the tested strains enables genetic association analysis and provide relatively small loci, facilitating the path to downstream QTG validation studies. The inbred mouse strains provide a unique renewable resource for *in vitro* screens as well as allow for downstream *in vivo* validation studies. Through follow-up studies, we have shown the utility of a screen using these cells to uncover a validated target in the *in vitro* toxicity of a chemical. Importantly, the findings from the screen were also shown to be translatable to a differential response in the whole animal. On a broader scale, the use of the approach enables a large number of compounds to be efficiently screened for the identification of putative toxicity pathways and core nodes in toxicity networks. Of note, this approach has also been shown in our lab to be applicable for other cell types (i.e., splenocytes), which could provide an innovative means for assessing specific toxicities for a particular class of compounds (Frick et al., 2013).

### Conflict of interest statement

The authors declare that the research was conducted in the absence of any commercial or financial relationships that could be construed as a potential conflict of interest.

## Supplementary material

Presentation 1 **(A)** Plate layout of compound concentrations and experimental controls used in the screen. Concentration ranges used for each compound can be found in Data Sheet 1. **(B)** MEF cells plating layout; each color represents a different mouse strain.

Data Sheet 1 **Tested compounds**.

Data Sheet 2 **Total number of data points removed from each drug-endpoint pair to allow for Brain-Cousens curve fitting**.

Data Sheet 3 **EC values used for genome-wide association analyses**. Compound concentrations are in nM and log transformed (base 10).

Data Sheet 4 **List of QTL and RefSeq genes with the associated drug, time point, and endpoint**.

Data Sheet 5 **Number of ECs used for each drug-endpoint pair at the two time points tested**. A maximum of seven ECs were obtained from the concentration-response curves, from EC_80_ to EC_50_ or EC_120_ to EC_150_, depending on the type of phenotypic response.

## References

[B1] Anilkumar N. Weber R. Zhang M. Brewer A. Shah A. M. (2008). Nox4 and nox2 NADPH oxidases mediate distinct cellular redox signaling responses to agonist stimulation. Arterioscler. Thromb. Vasc. Biol. 28, 1347–1354 10.1161/ATVBAHA.108.164277 18467643

[B2] Bedard K. Krause K. H. (2007). The NOX family of ROS-generating NADPH oxidases: physiology and pathophysiology. Physiol. Rev. 87, 245–313 10.1152/physrev.00044.2005 17237347

[B3] Benjamini Y. Hochberg Y. (1995). Controlling the false discovery rate: a practical and powerful approach to multiple testing. J. R. Stat. Soc. B 57, 289–300

[B4] Berg N. De Wever B. Fuchs H. W. Gaca M. Krul C. Roggen E. L. (2011). Toxicology in the 21st century–working our way towards a visionary reality. Toxicol. In Vitro 25, 874–881 10.1016/j.tiv.2011.02.008 21338664

[B5] Bleibel W. K. Duan S. Huang R. S. Kistner E. O. Shukla S. J. Wu X. (2009). Identification of genomic regions contributing to etoposide-induced cytotoxicity. Hum. Genet. 125, 173–180 10.1007/s00439-008-0607-4 19089452 PMC2714550

[B6] Bogue M. A. Grubb S. C. (2004). The mouse phenome project. Genetica 122, 71–74 10.1007/s10709-004-1438-4 15619963

[B7] Brain P. Cousens R. (1989). An equation to describe dose responses where there is stimulation of growth at low doses. Weed Res. 29, 93–96 10.1111/j.1365-3180.1989.tb00845.x

[B8] Chen G. Goeddel D. V. (2002). TNF-R1 signaling: a beautiful pathway. Science 296, 1634–1635 10.1126/science.1071924 12040173

[B9] Dang C. V. O'Donnell K. A. Zeller K. I. Nguyen T. Osthus R. C. Li F. (2006). The c-Myc target gene network. Semin. Cancer Biol. 16, 253–264 10.1016/j.semcancer.2006.07.014 16904903

[B10] Daniel P. B. Walker W. H. Habener J. F. (1998). Cyclic AMP signaling and gene regulation. Annu. Rev. Nutr. 18, 353–383 10.1146/annurev.nutr.18.1.353 9706229

[B11] Firestone M. Kavlock R. Zenick H. Kramer M. (2010). The U.S. Environmental Protection Agency strategic plan for evaluating the toxicity of chemicals. J. Toxicol. Environ. Health B Crit. Rev. 13, 139–162 10.1080/10937404.2010.483178 20574895

[B12] Frick A. Thomas R. Richards K. Damania B. Fedoriw Y. Parks B. (2013). Cellular genetics approaches to defining toxicity pathways, in AACR Annual Meeting 2013 (Washington, DC).

[B13] Gao H. M. Liu B. Hong J. S. (2003). Critical role for microglial NADPH oxidase in rotenone-induced degeneration of dopaminergic neurons. J. Neurosci. 23, 6181–6187 12867501 10.1523/JNEUROSCI.23-15-06181.2003 PMC6740554

[B14] Guo Y. Weller P. Farrell E. Cheung P. Fitch B. Clark D. (2006). *In silico* pharmacogenetics of warfarin metabolism. Nat. Biotechnol. 24, 531–536 10.1038/nbt1195 16680137 PMC1459533

[B15] Harrill A. H. Watkins P. B. Su S. Ross P. K. Harbourt D. E. Stylianou I. M. (2009). Mouse population-guided resequencing reveals that variants in CD44 contribute to acetaminophen-induced liver injury in humans. Genome Res. 19, 1507–1515 10.1101/gr.090241.108 19416960 PMC2752130

[B16] Higuchi H. Yamashita T. Yoshikawa H. Tohyama M. (2003). PKA phosphorylates the p75 receptor and regulates its localization to lipid rafts. EMBO J. 22, 1790–1800 10.1093/emboj/cdg177 12682012 PMC154469

[B17] Huang R. S. Duan S. Kistner E. O. Bleibel W. K. Delaney S. M. Fackenthal D. L. (2008). Genetic variants contributing to daunorubicin-induced cytotoxicity. Cancer Res. 68, 3161–3168 10.1158/0008-5472.CAN-07-6381 18451141 PMC2714371

[B18] Keller D. A. Juberg D. R. Catlin N. Farland W. H. Hess F. G. Wolf D. C. (2012). Identification and characterization of adverse effects in 21st century toxicology. Toxicol. Sci. 126, 291–297 10.1093/toxsci/kfr350 22262567 PMC3307604

[B19] Lee K. W. Zhao X. Im J. Y. Grosso H. Jang W. H. Chan T. W. (2012). Apoptosis signal-regulating kinase 1 mediates MPTP toxicity and regulates glial activation. PLoS ONE 7:e29935 10.1371/journal.pone.0029935 22253830 PMC3254627

[B20] Lim P. L. Liu J. Go M. L. Boelsterli U. A. (2008). The mitochondrial superoxide/thioredoxin-2/Ask1 signaling pathway is critically involved in troglitazone-induced cell injury to human hepatocytes. Toxicol. Sci. 101, 341–349 10.1093/toxsci/kfm273 17975114

[B21] Lock E. F. Abdo N. Huang R. Xia M. Kosyk O. O'Shea S. H. (2012). Quantitative high-throughput screening for chemical toxicity in a population-based *in vitro* model. Toxicol. Sci. 126, 578–588 10.1093/toxsci/kfs023 22268004 PMC3307611

[B22] Marshak D. R. Greenwalt D. E. (2007). Differentiating primary human cells in rapid-throughput discovery applications. Methods Mol. Biol. 356, 121–128 16988399 10.1385/1-59745-217-3:121

[B23] McClurg P. Janes J. Wu C. Delano D. L. Walker J. R. Batalov S. (2007). Genomewide association analysis in diverse inbred mice: power and population structure. Genetics 176, 675–683 10.1534/genetics.106.066241 17409088 PMC1893038

[B24] McClurg P. Pletcher M. T. Wiltshire T. Su A. I. (2006). Comparative analysis of haplotype association mapping algorithms. BMC Bioinformatics 7:61 10.1186/1471-2105-7-61 16466585 PMC1409800

[B25] Nako H. Kataoka K. Koibuchi N. Dong Y. F. Toyama K. Yamamoto E. (2012). Novel mechanism of angiotensin II-induced cardiac injury in hypertensive rats: the critical role of ASK1 and VEGF. Hypertens. Res. 35, 194–200 10.1038/hr.2011.175 22089532

[B26] National Research Council. Committee on Toxicity Testing and Assessment of Environmental Agents, A. (2007). Toxicity Testing in the 21st Century a Vision and a Strategy. Washington, DC: National Academies Press

[B27] Niso-Santano M. Gonzalez-Polo R. A. Bravo-San Pedro J. M. Gomez-Sanchez R. Lastres-Becker I. Ortiz-Ortiz M. A. (2010). Activation of apoptosis signal-regulating kinase 1 is a key factor in paraquat-induced cell death: modulation by the Nrf2/Trx axis. Free Radic. Biol. Med. 48, 1370–1381 10.1016/j.freeradbiomed.2010.02.024 20202476

[B28] O'Brien P. J. Irwin W. Diaz D. Howard-Cofield E. Krejsa C. M. Slaughter M. R. (2006). High concordance of drug-induced human hepatotoxicity with *in vitro* cytotoxicity measured in a novel cell-based model using high content screening. Arch. Toxicol. 80, 580–604 10.1007/s00204-006-0091-3 16598496

[B29] O'Shea S. H. Schwarz J. Kosyk O. Ross P. K. Ha M. J. Wright F. A. (2011). *In vitro* screening for population variability in chemical toxicity. Toxicol. Sci. 119, 398–407 10.1093/toxsci/kfq322 20952501 PMC3023564

[B30] Paigen K. Eppig J. T. (2000). A mouse phenome project. Mamm. Genome 11, 715–717 10.1007/s003350010152 10967127

[B31] Pal R. Monroe T. O. Palmieri M. Sardiello M. Rodney G. G. (2014). Rotenone induces neurotoxicity through Rac1-dependent activation of NADPH oxidase in SHSY-5Y cells. FEBS Lett. 588, 472–481 10.1016/j.febslet.2013.12.011 24374334 PMC3932663

[B32] Peters E. J. Kraja A. T. Lin S. J. Yen-Revollo J. L. Marsh S. Province M. A. (2009). Association of thymidylate synthase variants with 5-fluorouracil cytotoxicity. Pharmacogenet. Genomics 19, 399–401 10.1097/FPC.0b013e328329fdec 19339911

[B33] Rhind N. Russell P. (2012). Signaling pathways that regulate cell division. Cold Spring Harb. Perspect. Biol. 4:a005942 10.1101/cshperspect.a005942 23028116 PMC3475169

[B34] Scott C. W. Peters M. F. Dragan Y. P. (2013). Human induced pluripotent stem cells and their use in drug discovery for toxicity testing. Toxicol. Lett. 219, 49–58 10.1016/j.toxlet.2013.02.020 23470867

[B35] Seshacharyulu P. Pandey P. Datta K. Batra S. K. (2013). Phosphatase: PP2A structural importance, regulation and its aberrant expression in cancer. Cancer Lett. 335, 9–18 10.1016/j.canlet.2013.02.036 23454242 PMC3665613

[B36] Shanks N. Greek R. Greek J. (2009). Are animal models predictive for humans? Philos. Ethics Humanit. Med. PEHM 4:2 10.1186/1747-5341-4-2 19146696 PMC2642860

[B37] Silbergeld E. K. Contreras E. Q. Hartung T. Hirsch C. Hogberg H. Jachak A. C. (2011). t4 workshop report. Nanotoxicology: “the end of the beginning” - signs on the roadmap to a strategy for assuring the safe application and use of nanomaterials. ALTEX 28, 236–241 10.14573/altex.2011.3.236 21993959 PMC4038011

[B38] Venkatareddy M. Cook L. Abuarquob K. Verma R. Garg P. (2011). Nephrin regulates lamellipodia formation by assembling a protein complex that includes Ship2, filamin and lamellipodin. PLoS ONE 6:e28710 10.1371/journal.pone.0028710 22194892 PMC3237483

[B39] Venter J. C. Adams M. D. Myers E. W. Li P. W. Mural R. J. Sutton G. G. (2001). The sequence of the human genome. Science 291, 1304–1351 10.1126/science.1058040 11181995

[B40] Vinciguerra M. Foti M. (2006). PTEN and SHIP2 phosphoinositide phosphatases as negative regulators of insulin signalling. Arch. Physiol. Biochem. 112, 89–104 10.1080/13813450600711359 16931451

[B41] Wang N. Guan P. Zhang J. P. Chang Y. Z. Gu L. J. Hao F. K. (2011). Preventive effects of fasudil on adriamycin-induced cardiomyopathy: possible involvement of inhibition of RhoA/ROCK pathway. Food Chem. Toxicol. 49, 2975–2982 10.1016/j.fct.2011.06.080 21803115

[B42] Waterston R. H. Lindblad-Toh K. Birney E. Rogers J. Abril J. F. Agarwal P. (2002). Initial sequencing and comparative analysis of the mouse genome. Nature 420, 520–562 10.1038/nature01262 12466850

[B43] Watson V. G. Hardison N. E. Harris T. Motsinger-Reif A. McLeod H. L. (2011a). Genomic profiling in CEPH cell lines distinguishes between the camptothecins and indenoisoquinolines. Mol. Cancer Ther. 10, 1839–1845 10.1158/1535-7163.MCT-10-0872 21750217 PMC3191307

[B44] Watson V. G. Motsinger-Reif A. Hardison N. E. Peters E. J. Havener T. M. Everitt L. (2011b). Identification and replication of loci involved in camptothecin-induced cytotoxicity using CEPH pedigrees. PLoS ONE 6:e17561 10.1371/journal.pone.0017561 21573211 PMC3088663

[B45] Watters J. W. Kloss E. F. Link D. C. Graubert T. A. McLeod H. L. (2003). A mouse-based strategy for cyclophosphamide pharmacogenomic discovery. J. Appl. Physiol. 95, 1352–1360 10.1152/japplphysiol.00214.2003 12970373

[B46] Welsh M. Mangravite L. Medina M. W. Tantisira K. Zhang W. Huang R. S. (2009). Pharmacogenomic discovery using cell-based models. Pharmacol. Rev. 61, 413–429 10.1124/pr.109.001461 20038569 PMC2802425

[B47] Woodruff T. J. Zeise L. Axelrad D. A. Guyton K. Z. Janssen S. Miller M. (2008). Meeting report: moving upstream-evaluating adverse upstream end points for improved risk assessment and decision-making. Environ. Health Perspect. 116, 1568–1575 10.1289/ehp.11516 19057713 PMC2592280

[B48] Wu K. J. Mattioli M. Morse H. C. 3rd. Dalla-Favera R. (2002). c-MYC activates protein kinase A (PKA) by direct transcriptional activation of the PKA catalytic subunit beta (PKA-Cbeta) gene. Oncogene 21, 7872–7882 10.1038/sj.onc.1205986 12420224

[B49] Yang H. Ding Y. Hutchins L. N. Szatkiewicz J. Bell T. A. Paigen B. J. (2009). A customized and versatile high-density genotyping array for the mouse. Nat. Methods 6, 663–666 10.1038/nmeth.1359 19668205 PMC2735580

[B50] Yum J. Jeong H. M. Kim S. Seo J. W. Han Y. Lee K. Y. (2009). PKA-mediated stabilization of FoxH1 negatively regulates ERalpha activity. Mol. Cells 28, 67–71 10.1007/s10059-009-0099-7 19711044

[B51] Zhang X. Liu H. H. Weller P. Zheng M. Tao W. Wang J. (2011). *In silico* and *in vitro* pharmacogenetics: aldehyde oxidase rapidly metabolizes a p38 kinase inhibitor. Pharmacogenomics J. 11, 15–24 10.1038/tpj.2010.8 20177421

[B52] Zhou H. Zhang F. Chen S. H. Zhang D. Wilson B. Hong J. S. (2012). Rotenone activates phagocyte NADPH oxidase by binding to its membrane subunit gp91phox. Free Radic. Biol. Med. 52, 303–313 10.1016/j.freeradbiomed.2011.10.488 22094225 PMC3253173

[B53] Zwaenepoel K. Goris J. Erneux C. Parker P. J. Janssens V. (2010). Protein phosphatase 2A PR130/B″ alpha1 subunit binds to the SH2 domain-containing inositol polyphosphate 5-phosphatase 2 and prevents epidermal growth factor (EGF)-induced EGF receptor degradation sustaining EGF-mediated signaling. FASEB J. 24, 538–547 10.1096/fj.09-140228 19825976

