# Supplementary figures and images for: A cellular genetics approach identifies gene-drug interactions and pinpoints drug toxicity pathway nodes
Source: Front Genet. 2014 Aug 29;5:272. doi: 10.3389/fgene.2014.00272 (PMC4148776; doi:10.3389/fgene.2014.00272)

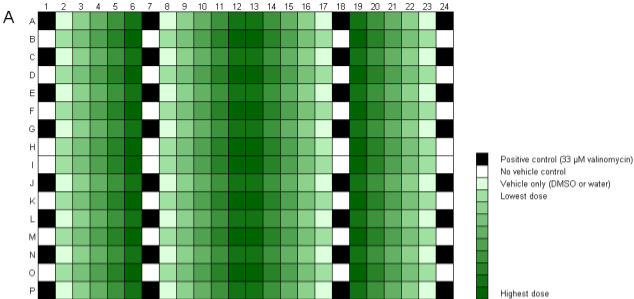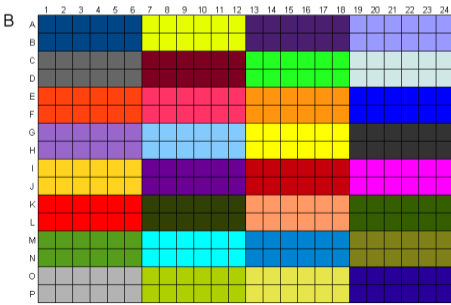

Supplement: Presentation 1 — (A) Plate layout of compound concentrations and experimental controls used in the screen. Concentration ranges used for each compound can be found in Data Sheet 1. (B) MEF cells plating layout; each color represents a different mouse strain. [file Presentation1.PDF]
